# Diaphragm adaptations in patients with COPD

**DOI:** 10.1186/1465-9921-9-12

**Published:** 2008-01-24

**Authors:** Coen AC Ottenheijm, Leo MA Heunks, Richard PN Dekhuijzen

**Affiliations:** 1 Dept. of Pulmonary Diseases, Radboud University Nijmegen Medical Centre, Nijmegen, The Netherlands; 2 Dept. of Intensive Care Medicine, Radboud University Nijmegen Medical Centre, Nijmegen, The Netherlands; 3 Institute for Fundamental and Clinical Human Movement Sciences, Radboud University Nijmegen Medical Centre, Nijmegen, The Netherlands; 4 Dept. of Molecular and Cellular Biology, University of Arizona, Tucson, USA

## Abstract

Inspiratory muscle weakness in patients with COPD is of major clinical relevance. For instance, maximum inspiratory pressure generation is an independent determinant of survival in severe COPD. Traditionally, inspiratory muscle weakness has been ascribed to hyperinflation-induced diaphragm shortening. However, more recently, invasive evaluation of diaphragm contractile function, structure, and biochemistry demonstrated that cellular and molecular alterations occur, of which several can be considered pathologic of nature. Whereas the fiber type shift towards oxidative type I fibers in COPD diaphragm is regarded beneficial, rendering the overloaded diaphragm more resistant to fatigue, the reduction of diaphragm fiber force generation *in vitro *likely contributes to diaphragm weakness. The reduced diaphragm force generation at single fiber level is associated with loss of myosin content in these fibers. Moreover, the diaphragm in COPD is exposed to oxidative stress and sarcomeric injury. This review postulates that the oxidative stress and sarcomeric injury activate proteolytic machinery, leading to contractile protein wasting and, consequently, loss of force generating capacity of diaphragm fibers in patients with COPD. Interestingly, several of these presumed pathologic alterations are already present early in the course of the disease (GOLD I/II), although these patients appear not limited in their daily life activities. Treatment of diaphragm dysfunction in COPD is complex since its etiology is unclear, but recent findings indicate the ubiquitin-proteasome pathway as a prime target to attenuate diaphragm wasting in COPD.

## Introduction

Chronic obstructive pulmonary disease (COPD) has been predicted to become the third leading cause of death and the fifth commonest cause of disability in the world by 2020 [1]. Today it is recognized that COPD affects function of other organs besides the lungs. In particular, dysfunction of the respiratory muscles has been shown to occur in patients with COPD. Dyspnea is the most disabling symptom in patients with COPD, and could result from a decreased capacity of the respiratory muscles to meet an increased mechanical load. Hypercapnic respiratory failure due to inspiratory muscle weakness [2] is associated with morbidity in these patients [3], and maximum inspiratory pressure is an independent determinant of survival in these patients [4]. These studies stress the importance of inspiratory muscle dysfunction in patients with COPD. Accordingly, the interest in defining the underlying causes of inspiratory muscle weakness in COPD has increased over the recent years.

The majority of studies dealing with factors contributing to inspiratory muscle weakness in COPD has focused on the diaphragm, mainly because the diaphragm is the principle muscle of inspiration. Patients with COPD have a lower transdiaphragmatic pressure generating capacity than healthy subjects [5], which has been ascribed to hyperinflation-induced diaphragm shortening, placing the diaphragm at a mechanical disadvantage [6]. When compensated for reduced muscle length, diaphragm function was suggested to be preserved or even improved in severe COPD [7]. However, the invasive evaluation of the human diaphragm has accelerated the understanding of the pathogenesis of diaphragm weakness in COPD. Since the late nineties an increasing number of papers has been published describing altered functional, structural and metabolic characteristics in diaphragm biopsies of patients with COPD [8-21]. Importantly, several of these changes occur already early in the course of the disease [11,12,18,19,21] and show a strong negative correlation with respiratory muscle strength [14,17]. Together these findings point towards a new concept in which the pathogenesis of inspiratory muscle dysfunction in COPD is related to cellular and molecular changes within the diaphragm, and occurs much earlier as previously assumed.

The aim of the present paper is to review the current knowledge on the effect of COPD on the human diaphragm, and to generate a hypothesis for further research. First, diaphragm performance *in vivo *will be discussed briefly. Second, studies describing changes in the diaphragm will be reviewed. Finally, potential causes for the observed cellular and molecular changes in COPD diaphragm will be addressed.

### Diaphragm performance in COPD: in vivo studies

Diaphragm performance is mainly characterized by its strength and endurance. Strength is defined as the capacity of the muscle to generate force and endurance is defined as the capacity of the muscle to maintain a certain force over time, in other words, to resist fatigue. Loss in either strength or endurance results in diaphragm weakness and impaired performance.

Force generation of the diaphragm *in vivo *is determined by central drive, phrenic nerve conductance, neuromuscular transmission and excitation-contraction coupling. Direct determination of diaphragm muscle force generating capacity *in vivo *is not possible. Instead the capacity of the inspiratory muscles to generate negative intrathoracic or transdiaphragmatic pressure is usually determined, and used as a measure for respiratory muscle strength. However, it should be emphasized that these methods do not solely depend on muscle function, but on motivation, central drive and nerve function as well.

It has been consistently demonstrated that patients with severe COPD generate less maximal inspiratory pressure and transdiaphragmatic pressure by voluntary manoeuvres, but also by phrenic nerve stimulation (ruling out effects of central drive), compared to patients without COPD [5,7,22-24]. Therefore, strength is regarded as a limiting factor in diaphragm performance in COPD. Indeed, inspiratory muscle weakness was shown to be related to dyspnea [25], and maximal inspiratory pressure is an independent determinant of survival in COPD [4]. Previous work by Similowski and co-workers [7] suggested that weakness of the diaphragm could be explained by hyperinflation-induced diaphragm shortening, which places the diaphragm on a suboptimal position on its force-length relationship. They revealed that at equivalent lung volumes, the generated transdiaphragmatic pressures were even higher in some patients with COPD compared to healthy subjects. However, in addition to a mechanical explanation for diaphragm weakness in COPD, several studies proposed structural adaptations in the diaphragm to be at play. For instance, parallel reductions of inspiratory and expiratory pressures are described in moderate-to-severe COPD patients [26,27]. Since the expiratory muscles are not at a mechanical disadvantage, these findings suggest that some COPD patients have generalized diaphragm weakness. This notion is supported by the high correlation between maximal inspiratory and expiratory pressures in their COPD patients [26].

The present data dealing with diaphragm endurance in COPD are limited, mainly because endurance tests are difficult to perform and interpret (for instance, diaphragm endurance tests imply normalized patient stimulation and a high degree of patient motivation). Newell et al. [5] tested diaphragm endurance by measuring pressure output during repeated maximal voluntary contractions of the inspiratory muscles. During the endurance sequence the decline in pressure was less in patients with severe COPD compared to individuals with normal lung function. Later, these findings were confirmed by other studies in patients with moderate-to-severe COPD [28,29]. So, despite the greater load and recruitment, diaphragm endurance appears to be increased in COPD. This is at least partly accounted for by diaphragm remodelling [15,16] and a shorter respiratory duty cycle [30]. For a review on this topic see Laghi and Tobin [6].

### Cellular and molecular changes in the diaphragm in COPD

In the last decade, several studies were published investigating diaphragm biopsies from both patients with and without COPD. The obtained diaphragm specimens allowed the evaluation of functional, structural and metabolic changes in diaphragm fibers of COPD patients. These studies improved the understanding of the effects of COPD on respiratory muscle performance.

#### Diaphragm fiber type distribution

A consistent alteration in the diaphragm of COPD patients is a fiber type shift towards more oxidative, type I, fibers.

Fiber typing can be performed histochemically by staining myofibrillar ATPase (type I, IIa, and IIx) [31] or by determination of mitochondrial enzyme activity (slow-twitch oxidative, fast-twitch oxidative-glycolytic, and fast-twith glycolytic) [32]. More recently, myofibrillar ATPase staining was shown to correspond to the expression of different myosin heavy chain isoforms (MHC_slow_, MHC_2A_, MHC_2B _and MHC_2X_), that can be identified by immunoreactivity to specific antibodies and/or by protein electrophoresis [33]. Type I fibers have a slow twitch, and depend mainly on aerobic metabolism and are therefore fatigue resistant. In contrast, type IIx fibers have a fast twitch and their metabolism is anaerobic glycolytic, which renders them susceptible for fatigue. Type IIa fibers have intermediate properties; they do have a fast twitch but are capable of working under both aerobic and anaerobic conditions and are therefore relatively fatigue-resistant. Maximal tension development of mammalian muscle is dependent on fiber type, with the lowest tension developed by type I fibers and the highest tension by type IIx fibers. However, in humans this fiber type-dependency of tension generating capacity is less obvious (for review see Bottinelli and Reggiani [34]).

**Figure 1:**
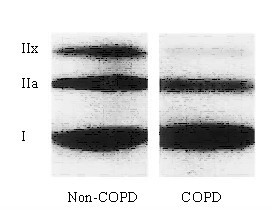
Myosin heavy chain isoform distribution, analyzed by western blot, in the costal diaphragm from severe COPD and non-COPD patients. Diaphragm from COPD patients contains a higher proportion of slow myosin heavy chain isoform and lower proportions of IIA and IIX isoforms compared to the diaphragm from non-COPD patients. Reproduced from Levine et al. [15] with permission.

The human diaphragm is chronically active and is among the most aerobicaly adapted striated muscles. Indeed, in individuals with normal lung function the diaphragm consists of a relatively high proportion (~50%) of type I fibers, compared to type IIa (~30%) and IIx (~20%) fibers [15,35,36]. In patients with COPD, the diaphragm works against an increased work load due to airflow limitation and geometrical changes in the thorax as a result of pulmonary hyperinflation. It has been hypothesized that the increased workload emulates endurance training of the diaphragm in these patients. In line with this, Levine and coworkers found an increased proportion of type I fibers in the diaphragm of patients with severe COPD, whereas the proportion of fast, fatiguing fibers (type II) was decreased [15] (figure 1). These findings were confirmed by other studies in patients with severe [13,17,35,37-39], but also mild-to-moderate COPD [19,21]. Several studies have shown that other inspiratory muscles, such as the external intercostal muscles, do not exhibit a fiber type shift in either direction in patients with moderate [40] and severe COPD [38]. The shift from type II towards type I fibers is regarded as beneficial as it may render the COPD diaphragm more resistant to fatigue [15].

Besides a shift towards type-I fibers, the diaphragm in severe COPD is characterized by a fast-to-slow shift of the sarcoplasmic reticulum calcium-ATPase (SERCA), as shown by Nguyen et al. [39]. In addition, the content of the fast SERCA-isoform was decreased. SERCAs pump calcium from the myoplasm into the sarcoplasmic reticulum and thereby initiate striated muscle relaxation. Although not studied, reduced SERCA pumping and the resulting slower relaxation could affect the force-frequency characteristics of diaphragm fibers in COPD. First, for a given level of force generation a lower stimulation frequency is needed. Second, lower stimulation frequencies will reduce the energetic cost of calcium pumping in COPD diaphragm fibers and thereby increase efficiency.

#### Diaphragm metabolism

Force generation by striated muscle is powered by adenosine triphosphate (ATP), a 'high energy' compound. Curiously, for such an important substance as ATP, it is found in relatively low concentrations within cells. Striated muscle, and especially the diaphragm with its high duty cycle, has a high metabolic rate and would use up the available ATP in a few seconds if it was not rapidly regenerated. ATP generation in striated muscle occurs via aerobic and anaerobic enzymatic pathways. *In vitro *determination of enzyme activities involved in muscle metabolism provides information regarding changes in expression of proteins involved in these metabolic pathways. Typical anaerobic enzymes are phophorylase, hexokinase and lactate dehydrogenase (LDH), which are mainly present in the cytosol. Typical aerobic enzymes are succinic dehydrogenase (SDH) citrate synthase (CS) and L(+)3-hydroacylCoA-dehydrogenase (HADH), which are mainly mitochondria-associated.

As mentioned previously, the different fiber types have distinct metabolic properties. Therefore, the shift towards type I fibers in COPD diaphragm is expected to result in an increased oxidative capacity. Indeed, it is well established that the activity of oxidative enzymes is increased in COPD diaphragm, with a concomitant decrease in glycolytic enzyme activities.

Several labs have reported increased activity of the oxidative enzymes CS, SDH and HADH in the diaphragm of patients with COPD [13,19,41], whereas the activities of the glycolytic enzymes hexokinase and LDH were decreased [42]. Similar to the fiber type shift, the changes in enzyme activities were present in some patients with only moderate COPD [19,42]. In addition to changes in enzyme activities, the mitochondrial oxidative capacity relative to ATP demand appears increased in the diaphragm of patients with severe COPD [13]. Also, enhanced function of the mitochondrial electron transport chain in the diaphragm of *severe *COPD patients has been reported [38]. Increased maximal mitochondrial respiration, and improved coupling of oxidation to phosphorylation was found in COPD diaphragm. In addition, these changes were associated with increase of CS activity. It was proposed that the increased mitochondrial efficiency in ATP production was the result of changed permeability of the inner mitochondrial membrane, thereby decreasing proton leak and basal respiration [38]. In patients with *moderate *COPD no changes in diaphragm mitochondrial efficiency were found [41] compared to healthy subjects.

Together these data indicate that the changes in oxidative capacity and mitochondrial function in the diaphragm occur in line with the progression of COPD and the concomitant shift towards more oxidative type I fibers. In other respiratory muscles, such as the external intercostals, the increase in oxidative capacity associated with COPD, appears to be less pronounced [38]. This was, at least in part, explained by the absence of a fiber type shift in these muscles.

#### Functional changes: diaphragm single fiber studies

##### Single fiber contractile properties

**Figure 2:**
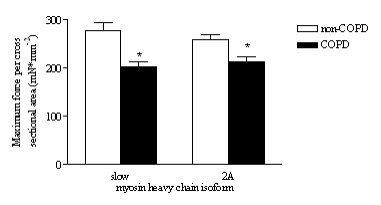
Maximum force generation of skinned diaphragm fibers from non-COPD and mild-to-moderate COPD patients. Maximum force, normalized to cross sectional area, of single fibers from COPD patients was lower in type slow and 2A fibers compared to non-COPD patients. Data are presented as model estimates ± sem. *: P < 0.05 different from non-COPD group. Reproduced from Ottenheijm et al. [18] with permission.

**Figure 3:**
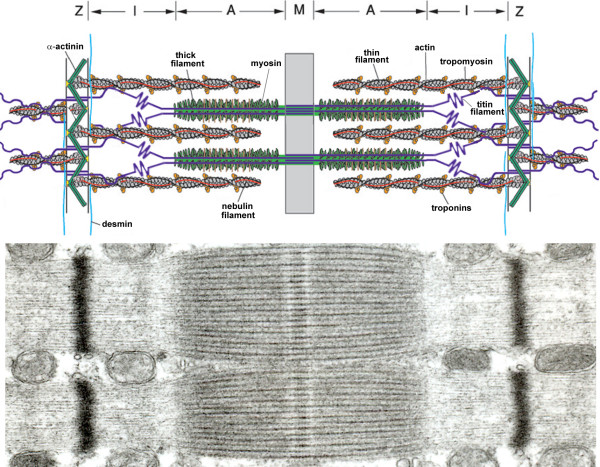
*Top*: Simplified model of two muscle sarcomeres in parallel. The sarcomere is comprised of the thin (mostly actin) filaments, the thick (mostly myosin) filaments, and the giant filamentous molecule titin. The thin filaments are anchored in the Z-line, where they are cross-linked by α-actinin. The thick filament is centrally located in the sarcomere and constitue the sarcomeric A-band. The myosin heads, or cross-bridges, on the thick filament interact with actin during activation. Titin spans the half-sarcomeric distance from the Z-line to the M-line, thus forming a third sarcomeric filament. In the I-band region, titin is extensible and functions as a molecular spring that develops passive tension upon stretch. In the A-band titin is inextensible due to its strong interaction with the thick filament.*Bottom*: Electronmiscroscopic photograph of the ultrastructural organization of sarcomeres in parallel.

Recent data from Levine and co-workers [14], and our group [18], indicate impaired contractile protein function in diaphragm fibers from patients with COPD. These studies evaluated the contractility of permeabilized (skinned) single fibers dissected from diaphragm biopsies obtained during thoracotomy. These biopsies do not allow dissection of muscle fibers from tendon to tendon. Thus fiber ends are not sealed, which disrupts normal excitation-contraction coupling. However, the skinned fiber model provides an excellent model for direct evaluation of contractile protein function in these fibers. In skinned fibers, the membranous structures, such as the sarcolemma, sarcoplasmic reticulum and mitochondria, are made highly permeable. This procedure leaves the contractile proteins intact. Therefore, a skinned fiber can be regarded as a collection of functionally isolated sarcomeres in series and in parallel. Mounting of skinned fibers between a force transducer and a length motor and subsequent exposure of the fiber to calcium enables evaluation of contractile protein function. Recent data from our lab [18] showed markedly reduced maximum force generation of diaphragm fibers from patients with only mild-to-moderate COPD compared to non-COPD patients. (figure 2) The maximum force generated by a muscle fiber is strongly dependent on the content of myosin, the main contractile protein (figure 3). The reduction of maximum force generated by COPD diaphragm fibers, as observed in our study, was associated with loss of myosin content in these fibers [18]. On the basis of their single fiber data, Levine et al. [15] calculated that patients with severe COPD would generate only 60% of the average maximal transdiaphragmatic specific force compared to individuals with normal lung function. Because maximal transdiaphragmatic pressures of severe COPD patients are reduced to approximately 65% of control values [7,22-24], they proposed that changes at the cellular and molecular level in the diaphragm of COPD patients can fully account for the reduced diaphragm strength *in vivo *in these patients [14].

It should be noted however, that *in vivo *the diaphragm shortens against a *submaximal *load rather than that it performs maximum isometric contractions. Thus, submaximal and kinetic parameters of muscle function provide important physiological information. We found that at a certain calcium concentration, the generated force relative to maximum force, is less in fibers from COPD patients compared to non-COPD patients [18]. In other words, the calcium sensitivity of force generation is reduced. This appears to be an important finding, as it could compromise diaphragm function at submaximum activation *in vivo*. Moreover, diaphragm fibers from COPD patients had slower attachment/detachment rates of myosin to actin during activation [18]. These data show that, besides loss of contractile protein in the diaphragm of patients with mild-to-moderate COPD, the remaining contractile proteins are dysfunctional. The molecular basis of this protein dysfunction is unclear. However, probably myosin and troponin are involved, as the functioning of these proteins is an important determinant of myosin-actin attachment/detachment rates and calcium sensitivity of force generation.

##### Single fiber passive properties

**Figure 4:**
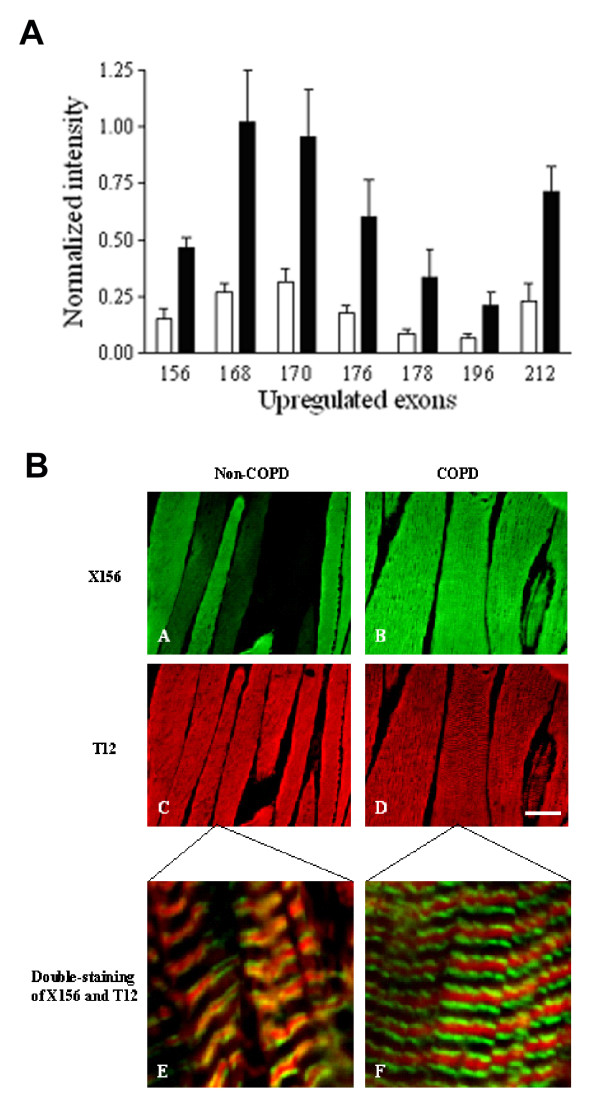
A: Analysis of diaphragm transcripts of all titin's gene exons from mild-to-moderate COPD and non-COPD patients. All exons listed are upregulated by at least 3-fold (*P *< 0.05) in diaphragm from COPD patients (black bars) when compared with diaphragm from non-COPD patients (white bars), and code for the extensible I-band segment of titin (i.e. PEVK segment). B: Immuno-fluorescence analysis of antibody ×156, directed against the titin domain encoded by exon 156, and antibody T12, directed against a titin domain near the sarcomeric Z-line. Double-staining with antibodies ×156 and T12 revealed increased staining intensity of ×156 in diaphragm cryosections from patients with COPD (green, A-B), whereas staining intensity of T12 was comparable between both patient groups (red, C-D). Bar: 50 μm. Confocal microscopy demonstrated the expected staining pattern of both antibodies: red striation patterns of T12 indicating the location of the sarcomeric Z-lines and green striations of ×156 indicating the extensible titin regions of the sarcomere (E-F). Bar: 2.5 μm. Reproduced from Ottenheijm et al. [21] with permission.

For optimal contractile function, besides contractile proteins such as myosin, passive-elastic structures in the sarcomere are needed [43]; deletion of these structures abolishes both passive and active force generation by single muscle fibers [43]. Therefore, in addition to active force generation, passive force characteristics of muscle fibers provide important information on muscle function. Recently, we [21], and others [12], demonstrated that diaphragm fibers from patients with mild-to-moderate COPD generate less passive tension upon fiber-stretch compared to fibers from patients without COPD. Passive tension in these fibers is mainly determined by the properties of titin [44,45]. Titin spans the half-sarcomeric distance from the Z-line to the M-line, thus forming a third sarcomeric filament, apart from the thick (mostly myosin) and thin (mostly actin) filaments [46]. In the I-band region, titin is extensible (due to its elastic PEVK segment) and functions as a molecular spring that develops passive tension upon stretch. Titin gene transcript studies revealed increased expression of exons coding for the spring elements in the elastic region of titin in COPD diaphragm [21], most likely resulting in an elongated extensible titin segment. (figure 4) These data strongly suggest that alternative splicing of the titin gene reduces the passive tension generated by diaphragm fibers from COPD patients. In contrast to myosin and nebulin content, titin content was not reduced in the diaphragm of these patients. Apparently, the diaphragm of patients with mild-to-moderate COPD is subject to loss of specific proteins and qualitative changes within molecules.

#### Diaphragm atrophy

Muscle atrophy is defined as the wasting or loss of muscle tissue, resulting from an imbalance between protein synthesis and degradation due to disease or deconditioning. Atrophy is characterized by a decrease in protein content and fiber cross sectional area resulting in reduced force production. Diaphragm fiber atrophy is a consistent finding in patients with severe COPD.

Both type I and type II diaphragm fibers show reduced cross sectional area in patients with severe COPD compared to non-COPD patients [15,47]. Another study in severe COPD patients found reduced cross sectional area in predominantly type I diaphragm fibers [13]. In contrast to patients with severe COPD, reductions in diaphragm fiber cross sectional area have not been found in mild or moderate COPD patients [16,19,20,48,49]. Although these data suggest that fiber atrophy occurs only in severe COPD, measuring cross sectional area is not a very sensitive method for detection of atrophy. Indeed, recent data show contractile protein loss in the diaphragm in mild-to-moderate COPD [11,18,21] (FEV_1 _~70% predicted). In these patients, the myosin content in both type I and IIa diaphragm fibers was markedly reduced, while fiber cross sectional area remained normal. Consequently, myosin concentration was decreased in these fibers by ~50% [18]. As expected, this reduction in myosin content was associated with decreased maximum force generation in those fibers, as discussed earlier.

In general, striated muscle atrophy results from increased proteolysis, rather than decreased synthesis [50]. During atrophy the bulk of myofibrillar protein degradation occurs via the ubiquitin-proteasome pathway [51,52]. Proteolysis by the ubiquitin-proteasome pathway is highly selective and precisely regulated [53]. Proteins degraded via this pathway are labelled first with ubiquitin through the action of specific ubiquitin ligases. In general, only damaged or misfolded proteins are labelled. Ubiquitin-conjugated proteins are subsequently recognized, bound and degraded by the 20S proteasome [51]. Recent work demonstrated activation of the ubiquitin-proteasome pathway in the diaphragm of patients with mild-to-moderate COPD, as indicated by increased proteasome activity, elevated mRNA levels of the ubiquitin ligase MAFbx [11], and elevated levels of ubiquitin-conjugated proteins [18]. Moreover, unpublished data revealed that inhibiting proteasome activity *in vivo *in an animal model of COPD partially restored diaphragm myosin content and force generation capacity [54]. These findings strongly suggest a key role for ubiquitin-proteasome-mediated muscle protein degradation in diaphragm weakness in COPD. Activation of the endoprotease caspase-3 is an initial step in myofilament proteolysis by cleavage of myosin and actin [55]. In this way, activated caspase-3 yields fragments that are degradable by the ubiquitin-proteasome pathway. Indeed, caspase-3 activity is increased in the diaphragm of patients with mild-to-moderate COPD compared to non-COPD patients [11].

Little is known regarding mediators that affect the rate of muscle protein synthesis in COPD diaphragm, although glycosaminoglycans might be involved. Glycosaminoglycans are linear unbranched polysaccharides, most of which are covalently linked to a protein core to form proteoglycans [56]. Depending on the nature of the glycosaminoglycan-moiety, one can discern heparan sulfate, dermatan sulfate, and chondroitin sulfate proteoglycans. Most proteoglycans are found either on the cell surface, or in the extracellular matrix [57,58]. In skeletal muscle glycosaminoglycans are involved in numerous biological processes, notably the orchestration of anabolic and catabolic signaling by unique sulfation patterns on the heparan sulfate molecule (for review see [59]). Heparan sulfate is essential for the activation of individual members of several growth factor families, including hepatocyte growth factor and insulin-like growth factor [60-65]. The dynamic spatiotemporal expression of proteoglycans and heparan sulfate epitopes provides a micro-environment in which heparan sulfate mediates growth factor activity by creating focal differences in concentration and by facilitating ligand-receptor interactions [66]. Through this modulating effect on growth factors, glycosaminoglycans are instrumental in skeletal muscle protein synthesis and regeneration [67-69]. Recent work [10] indicates down-regulation of a specific heparan sulfate epitope in the diaphragm of patients with COPD. This down-regulated heparan sulfate epitope appeared to be involved in the binding of hepatocyte growth factor. As hepatocyte growth factor is an important modulator of contractile protein synthesis [70], its down-regulation may affect contractile protein content in the diaphragm of patients with COPD. Interestingly, these changes already occur in patients with mild-to-moderate COPD (GOLD stage I/II).

It should be noted that the reduced diaphragm fiber cross sectional area in severe COPD, besides compromising diaphragm force generation, could also represent a beneficial adaptation by facilitating oxygen transport and diffusion from the capillaries into the fibers to meet the increased oxidative metabolism in COPD diaphragm (as discussed earlier). However, previous studies reported comparable capillarization between individuals with normal lung function and mild-to-moderate [19] and moderate-to-severe COPD patients [17], although diaphragmatic mRNA levels of vascular endothelial growth factor are increased in patients with moderate COPD [71], suggesting enhanced angiogenesis.

#### Diaphragm injury

Striated muscle injury is characterized by morphological abnormalities such as disruption of membranous structures (sarcolemma, mitochondria etc), degeneration of the cytoplasm, and disorganization of the contractile apparatus (including sarcomere disruption, Z-line streaming and misalignment of the myofilaments). It is well established that exertion can have profound effects on striated muscle structure and can induce muscle injury. For instance, injury of the diaphragm has been observed in several animal models of respiratory loading [72,73]. Recent data clearly demonstrate increased diaphragm injury in patients with COPD.

**Figure 5:**
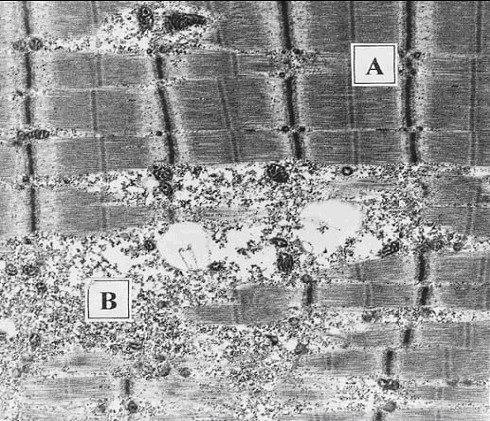
Electronmicroscopic photograph showing areas of normal (A) and disrupted (B) sarcomeres in a diaphragm sample from a patient with moderate-severe COPD. Note the disruption and even absence of A- and I-bands. Reproduced from Orozco-levi et al. [20] with permission.

Increased sarcomere disruption has been found in the diaphragm of patients with moderate-to-severe COPD [8,20] (figure 5). The FEV_1 _was inversely correlated with both sarcomere disruption density and area fraction [20]. Data from our group show Z-band streaming and accumulations of Z-band material in the diaphragm, indicating myofibrillar myopathy, in severe COPD [74]. In another study in mild-to-moderate COPD patients (average FEV_1_, 60% predicted), diaphragm cross sections showed no signs of injury, although sarcomere length appeared shorter [16]. The latter was proposed to be the result of hyperinflation-induced diaphragm shortening. Interestingly, diaphragms from patients with moderate-to-severe COPD were found to be three times more susceptible to additional sarcomere disruption when breathing against inspiratory loads compared to non-COPD patients [20]. In line with these findings, recent data from a *post mortem *study in severe COPD patients reveal that acute-on-chronic increase in ventilatory loading induced extensive diaphragm injury and collagen accumulation [48]. Whereas intracellular, sarcomeric, injury in COPD diaphragm is evident, the membrane and the membrane-associated proteins, which are essential for force transmission to adjacent fibers, appear intact [75]. Together, these data indicate that sarcomeric injury is more pronounced in COPD diaphragm and increases with progression of the disease.

In general, muscle injury is followed by an inflammatory response and subsequent regeneration. However, previous studies found no evidence of inflammatory cells in the diaphragm of patients with COPD, although injury was evident, suggesting an altered response to injury [8,20]. Muscle degeneration is followed by the activation of a muscle repair or regeneration process in which satellite cell activation is an important step [70]. Activated satellite cells proliferate and differentiate to new myofibers. These newly formed fibers express embryonic/neonatal forms of myosin heavy chain [70]. However, preliminary data from our lab indicate that diaphragm satellite cell differentiation into myotubes is impaired in COPD [76]. Also, Nguyen et al. [37] found lower expression of embryonic/neonatal myosin heavy chains in the diaphragm from COPD patients compared to non-COPD patients, and similar findings were reported in a preliminary study from our lab [77]. Finally, in an animal model of COPD, there was evidence of decreased MyoD and elevated inhibitor of differentiation protein 2 (Id2) levels in diaphragm muscle [78], which may impair satellite cell differentiation, and thus its regenerative capacity. Together, these data suggest a non-adequate response to diaphragm injury in patients with COPD.

#### Oxidative and nitrosative stress

Oxidative and nitrosative stress reflect an imbalance between the production and scavenging of free radicals. Free radicals are molecules with at least one unpaired electron in their outer orbital. Since electrons are usually more stable when paired, radicals are more reactive than non-radicals. *In vivo*, free radicals can be divided into oxygen- and nitrogen-centred free radicals, such as superoxide anion and nitric oxide, respectively. Sources for free radicals in striated muscle include the mitochondrial electron transport chain, xanthine oxidase, neutrophils and nitric oxide synthases. Striated muscles generate free radicals at rest and production increases during contractile activity [79,80]. Their high reactivity makes it technically extremely complex to measure free radical levels inside muscle fibers. Therefore, in general, oxidative stress is determined indirectly by measuring the levels of oxidized proteins and/or lipids.

A recent study by Barreiro et al. [17] was the first to demonstrate increased oxidative stress in the diaphragm of patients with severe COPD. They reported higher protein carbonyl groups and hydroxynonenal protein adducts, both markers of protein oxidation. Importantly, the diaphragmatic levels of protein oxidation showed a strong negative correlation with respiratory muscle strength. In the diaphragm of moderate COPD patients no signs of oxidative stress were present. In contrast to oxidative stress, nitrosative stress was not elevated in COPD diaphragm [17]. This was supported by unchanged protein tyrosine nitration levels in the diaphragm of these patients.

### Diaphragm versus peripheral muscles in COPD

One of the most remarkable alterations in COPD muscle is the fiber type shift that occurs in opposite directions in the diaphragm and peripheral muscles. Whereas the fiber type shift in the diaphragm resembles that associated with endurance training, adaptation of the limb muscles is characterized by a shift towards more fatigable, glycolytic type II fibers in severe COPD [81-84]. The changes in fiber type composition of peripheral muscles in COPD correlate with the reduced activity of oxidative enzymes in those muscles [85,86]. A study by Doucet et al. was the first to investigate fiber type proportions in the diaphragm and a limb muscle within the same patients with mild-to-moderate COPD. They found a fiber type shift in the diaphragm, whereas a fiber type shift in limb muscles was absent in these patients [19]. Apparently, fiber type shifts in the diaphragm occur earlier in the disease process than the changes in the limb muscles. In addition, several other abnormalities associated with COPD are specific for peripheral muscles or the diaphragm. For instance, in contrast to the diaphragm, peripheral muscles in COPD associated with low body-mass-index show accelerated apoptosis and inflammatory changes [87]. Also, whereas diaphragm injury is a common feature in severe COPD, profound peripheral muscle injury has not been reported.

On the other hand, atrophy appears to be a common feature in both the diaphragm and peripheral muscles of COPD patients [81,88]. Also, similar to the diaphragm, the peripheral muscles in COPD are exposed to oxidative stress [89-92]. Nitrosative stress, however, appears to be more specific to the peripheral muscles than the diaphragm, and was proposed to be the result of increased activity of neuronal nitric oxide synthase in peripheral muscles of COPD patients [92-94].

It should be noted that the concept of peripheral muscles needs some differentiation. In general, the structure and function of the upper limb muscles appears to be less affected in COPD, presumably due to sustained activity during daily activities, whereas muscles from the lower limbs show more pronounced changes as a result of progressive deconditioning [95].

### Genesis of a pathophysiological concept

The fiber type shift towards more oxidative type I fibers may initially be regarded as beneficial as it renders the overloaded COPD diaphragm more resistant to fatigue [15]. In fact, contractile fatigue is an uncommon event after exhaustive exercise in severe COPD [5,29,96]. However, the loss of myosin and force generating capacity in diaphragm single fibers, and the increased sarcomeric injury and oxidative stress, constitute maladaptive alterations in the diaphragm of these patients [8,14,17,18,20], and have been suggested to greatly impair diaphragm function *in vivo *[14,17]. Together, these findings constitute a cellular and molecular basis for the the notion that strength and not fatigue is the main limiting factor for *in vivo *respiratory muscle performance in COPD.

The strength of the diaphragm is strongly dependent on its contractile protein content. Recent data suggest that accelerated protein degradation via the ubiquitin-proteasome pathway contributes to loss of myosin in COPD diaphragm, and constitutes an initial step in the pathogenesis of diaphragm weakness in these patients. Chamberlain [97] addressed the role of selective degradation of myosin by the ubiquitin-proteasome pathway in cachexia [98]. Preferential loss of myosin would increase the average distance between myosin and actin filaments, inducing reduction of fiber size later in the course of the disease to restore filament lattice spacing needed for optimal contractile function [97]. Indeed, loss of myosin precedes reduction of fiber cross sectional area in COPD diaphragm [18]. Although speculative, these findings support the hypothesis that, in mild-to-moderate COPD, loss of myosin is an initial, but functional important, step in diaphragm muscle fiber atrophy, as observed in severe COPD.

The giant protein titin might also be involved in diaphragm atrophy in COPD. The passive-elastic properties of titin are essential for maintaining structural and mechanical stability of the sarcomere during activation [43,99]. However, recent studies indicate that titin is an important player in the maintenance of striated muscle mass as well [100]. Titin functions as a stretch sensor with titin-based stiffness regulating muscle remodelling and gene expression, through a signaling pathway linking activity of titin's kinase domain to nuclear transcriptional activity [100]. Reduced mechanical strain on the titin kinase domain resulted in down-regulation of muscle-specific gene transcription, and was suggested to cause loss of contractile protein and muscle weakness in patients with hereditary myopathy with early respiratory failure [100]. We hypothesize that the reduced stiffness of titin in the diaphragm of mild-to-moderate COPD patients reduces its kinase domain activity, thereby inducing loss of signaling between titin and the nucleus, resulting in decreased transcription of muscle-specific genes. This could affect the production of contractile proteins, and contribute to the loss of myosin in the diaphragm of these patients, especially since diaphragm protein turnover might be elevated.

The increased susceptibility of the COPD diaphragm to inspiratory loading-induced sarcomeric injury appears to be an important finding as it suggests that during exacerbations of COPD, when patients experience acute diaphragm loading, the diaphragm may develop additional injury. This could compromise diaphragm strength even more and consequently result in respiratory failure. Indeed, COPD patients experiencing acute-on chronic respiratory loading have extensive diaphragm fiber injury and fibrosis[48]. Moreover, the increased susceptibility to injury implies that, although the sarcomeric ultrastructure appears intact during electronmiscroscopical examination, the structural integrity of the sarcomere is, in fact, weakened. Reduced stiffness of titin in COPD diaphragm might play a role, as it could induce structural instability of sarcomeres, leading to misalignment of myosin filaments and inability of the muscle fiber to resist sarcomere length inhomogeneity during activation. This overstretching of sarcomeres could contribute to the sarcomeric injury observed in the diaphragm of patients with severe COPD. In addition, the elevated levels of oxidized sarcomeric proteins in the diaphragm of patients with severe COPD might contribute to the increased susceptibility to diaphragm injury. Oxidized proteins have impaired structural integrity and function, and are known substrates for degradation via the ubiquitin-proteasome pathway [101]. These subtle structural modifications might be already present in patients with mild-to-moderate COPD, and play a role in the observed contractile protein dysfunction and the pronounced loss of myosin by accelerating ubiquitin-proteasome-mediated proteolysis. However, this is not supported by previous findings in moderate COPD patients, indicating similar levels of oxidized diaphragm proteins compared to non-COPD patients [17]. The detection of oxidatively modified proteins is, however, dependent on the sensitivity of the assays to measure such damage [102]. Therefore, future studies using highly sensitive proteomic analysis could resolve this issue. In healthy striated muscle, upon injury a rapid and extensive repair process is initiated to prevent loss of muscle mass. The fact that COPD diaphragm displays impaired regenerative processes, although injury is evident, suggests that impaired regeneration is involved in diaphragm atrophy.

The knowledge regarding diaphragm protein synthesis in COPD is very limited. Therefore, a major challenge for future studies will be to evaluate the relative contributions of protein degradation and synthesis pathways to the loss of contractile protein content in COPD diaphragm.

### Identifying initial triggers leading to diaphragm weakness in COPD

Little is known regarding the etiology of cellular and molecular changes in COPD diaphragm. Therefore, a major challenge for future studies will be to identify the triggers responsible for the observed changes in the COPD diaphragm. This is especially challenging since several adaptations are already present early in the course of the disease. Mechanisms that might be involved include non-systemic factors, such as increased diaphragm loading due to pulmonary obstruction, and sytemic factors, such as systemic inflammation. However, no studies have reported systemic changes in patients with mild-to-moderate COPD. Also, if a systemic etiology is proposed, alterations in the diaphragm and peripheral muscles are expected to share a high degree of similarity and, to a certain extent, develop simultaneously. However, the diaphragm and peripheral muscles appear differentially affected in COPD, and the diaphragmatic alterations appear to precede the peripheral muscle changes. Therefore, at early onset of the disease, most likely other factors are involved in the etiology of diaphragm weakness.

A potential trigger for the diaphragmatic changes in COPD patients is elevated contractile activity of the diaphragm. Loaded breathing is known to impair diaphragm contractility in healthy humans [103]. It was proposed that free radicals were involved since treatment with *N*-acteyl-cysteine, a molecule with antioxidant properties, attenuated diaphragm dysfunction in these subjects [103]. In patients with severe COPD, diaphragm loading is increased [104], and recent data suggest oxidative stress in the diaphragm of these patients. Very little is known about diaphragm loading in patients with only mild-to-moderate COPD. Markers for oxidative stress were not elevated in the diaphragm of these patients, but oxidative protein modification could be too subtle for detection with the applied assays. The fiber type shift towards type I fibers in some patients with mild-to-moderate COPD does suggest elevated contractile activity of the diaphragm in these patients. With this in mind, we stress the importance of future studies aimed at evaluating diaphragm contractile activity in patients with mild or moderate COPD. The obtained data should indicate whether or not increased diaphragm loading might play a role in the development of diaphragm weakness in COPD.

Cytokines promote striated muscle injury, impair contractile protein function [105] and stimulate proteolysis through the ubiquitin-proteasome pathway *in vitro *[106-108]. Circulating cytokines are proposed to initiate cachexia-associated muscle wasting by selective targeting myosin [97]. At rest, circulating levels of cytokines are elevated in COPD, but only in a subgroup of severe COPD patients [109-111]. However, physical exercise induces an exaggerated systemic inflammatory and oxidative response in patients with moderate COPD [112]. This abnormal response could be harmful by inducing skeletal and diaphragm muscle changes. It would be interesting to know if this abnormal exercise-induced inflammatory response is also present in patients with mild-to-moderate COPD. In addition, the source for the exercise-induced cytokines are yet to be determined. They have been attributed to bronchial inflammation, however, recent data indicate differential regulation of the cytokine response in induced sputum and plasma in COPD patients [113], which may suggest additional origins for COPD-associated cytokinemia. The diaphragm provides an alternative candiate. Diaphragm loading induces expression of cytokines in rat diaphragm fibers [114] (for review see Vasillakopoulos et al[115]). We speculate that the expression of cytokines is increased in the diaphragm of patients with mild-to-moderate COPD, contributing to the observed diaphragmatic changes by promoting sarcomeric injury, and protein modification and degradation. Therefore, evaluation of the cytokine profile in the diaphragm of patients with mild or moderate COPD will increase our understanding of the etiology of the cellular and molecular changes in COPD diaphragm.
